# Identifying sexual behavioural choices when commencing pre-exposure prophylaxis: Findings from a public health facility in eThekwini, KwaZulu-Natal, South Africa

**DOI:** 10.4102/sajhivmed.v27i1.1758

**Published:** 2026-02-04

**Authors:** Jyotika Basdav, Poovendhree Reddy, Firoza Haffejee

**Affiliations:** 1 Department of Basic Medical Sciences, Faculty of Health Sciences, Durban University of Technology, Durban, South Africa; 2 Department of Community Health Studies, Faculty of Health Sciences, Durban University of Technology, Durban, South Africa

**Keywords:** sexual behavioural choices, pre-exposure prophylaxis initiation, self-perceived HIV risk, HIV risk profile, KwaZulu-Natal, South Africa

## Abstract

**Background:**

HIV/AIDS continues to be of concern, particularly in South Africa, where the current prevalence is 12.7%.

**Objectives:**

This study aimed to explore sexual practices that increased HIV vulnerability among clinic users who chose to initiate pre-exposure prophylaxis (PrEP) in KwaZulu-Natal, South Africa.

**Method:**

A quantitative approach was utilised to collect data at an urban clinic in eThekwini (*n* = 376). Data were analysed using SPSS v29.

**Results:**

Participants were predominantly female (*n* = 331; 88%), single (*n* = 343; 91.5%), and heterosexual (*n* = 368; 98.1%). Sexual debut occurred at 18.4 ± 2.58 years. More men reported having concurrent sexual partners (*n* = 24; 54.5%). Transactional sex was more often reported by single women aged 18 years to 24 years. Age-disparate relationships were more common among those aged 18 years to 24 years. A significant association between early sexual debut and the number of lifetime partners was identified (*P* < 0.001).

**Conclusion:**

Our findings indicate that the study population was aware of their increased HIV vulnerability and were willing to try preventive measures, such as PrEP, to protect against seroconversion. However, interventions are required to abate the cycle of transactional sex, age-disparate relationships, and early sexual debut, especially among young women aged 15 years to 24 years.

**What this study adds:** The study identified that participants were aware of their self-perceived HIV risk and were willing to try PrEP. The driving factors for their high HIV risk were transactional sex, age-disparate relationships, and early sexual debut.

## Introduction

Vulnerable populations, such as adolescent girls and young women, orphans, street children, serodiscordant couples, and migrant workers, are at high risk of contracting HIV, based on their sexual behavioural practices.^1,2^ In addition, there are a multitude of interlinking risk factors that increase one’s chance of acquiring HIV, including early sexual debut, multiple concurrent sexual partners, lack of condom use, sexually transmitted infections, and transactional sex. Early sexual debut (at 15 years or younger) was reported across both genders in South Africa,^3,4,5,6,7^ but is more frequent amongst boys aged 14 years or younger,^3,8^ and commonly reported among those residing in urban locations.^9^ However, non-consensual sex is reported by girls as young as 13 years, many of whom were coerced by older men,^5^ placing them at a greater risk of contracting HIV^4^ and sexually transmitted infections.^5^ Age-disparate relationships are defined as a sexual partner who was at least 5 years older than the participant, and this increases the chance of HIV acquisition because of the difficulty in negotiating safe sexual practices.^6^

These age-disparate relationships are often transactional in nature, with the female partner viewing the receipt of material items as an expression of love.^10^ These young women are at high risk of HIV acquisition as they do not have the ability to negotiate safe sexual activity.^10,11,12^ Moreover, multiple concurrent relationships of a transactional nature were reported among a cohort of young women in the North West province of South Africa,^11^ as this increased their chances of monetary and material gain.^11,12^

Other factors, such as education inequalities and informal housing, contribute to sexual behaviour resulting in high HIV transmission rates.^13,14,15^ Psychosocial factors, such as trauma exposure and lack of emotional support, negatively impact their attitudes towards relationships and sex. Lack of advice and support systems usually contributes to their views on relationships and sex.^16^ A study conducted on adolescent girls and young women in the South African provinces of KwaZulu-Natal and Gauteng found an association with depressive symptoms, substance use, and intimate partner violence that increased their risk of HIV acquisition.^17^ Another study conducted in Cape Town, South Africa, highlighted that women and girls from a lower socio-economic background were more likely to contract HIV compared to their male counterparts. Women and girls were less likely to use a condom or have multiple partners.^15^ Transactional sex is a key factor in HIV, as this results in age-disparate relationships and unequal gender dynamics in relationships. Men were seen as financial benefactors and, under these circumstances, the women and girls were unable to negotiate safe sexual practices. Furthermore, material and economic gains outweighed the risk of HIV. These factors underscore the need to empower women economically, to explore innovative ways to market HIV prevention methods, and to address cultural norms.

A study conducted by Madiba and Ngwenya^18^ in the Mpumalanga province of South Africa highlighted women’s fear of physical and sexual violence when they suggested condom use to their partners. These women feared that their partners would discuss these matters among family members, feared the relationship would end in divorce, and assumed they would be subjected to stigma. Men paid a bride price (lobola) and felt they were entitled to dictate sexual practices within the relationship. Under these circumstances, men were assumed to be dominant while women were submissive, especially because of their financial dependence on their partners.^18^

Existing interventions, such as condom use, male circumcision, harm reduction programmes for drug users, and elimination of mother-to-child transmission,^19^ are currently used as part of HIV prevention strategies; however, the HIV epidemic continues unabated in South Africa. In 2016, pre-exposure prophylaxis (PrEP), an oral tablet taken daily, was introduced for HIV prevention in high-risk populations. Pre-exposure prophylaxis can be used during high-risk exposure stages and phased off when no longer required,^20^ and can be used in conjunction with other biomedical interventions. Studies have highlighted the importance of PrEP initiation in populations with poor condom negotiation skills, transactional sex, concurrent sexual relationships, and serodiscordant relationships.^21,22,23,24^ The success of this prophylaxis will be determined by one’s awareness of their HIV risk exposure. However, a recent study reported very low awareness of PrEP among young people, and users of a primary healthcare clinic in KwaZulu-Natal.^25^ The current study was located in an urban clinic, where PrEP was integrated into existing sexual reproductive health services as part of the healthcare facility-based model. This study aimed to offer PrEP to those at risk of acquiring HIV, and explored the baseline sexual practices that increase HIV vulnerability of the participants who enrolled for PrEP at a KwaZulu-Natal primary healthcare clinic offering reproductive health services.

## Research methods and design

This quantitative study was conducted at a primary healthcare clinic located in the central business district in eThekwini, KwaZulu-Natal, South Africa. Data were collected from January 2022 to April 2023, using purposive sampling. This type of sampling allows one to gain knowledge about a particular phenomenon. Greater preference is given to the knowledge aspect rather than the statistical outcome of the research. Purposive sampling is beneficial in explanatory studies, and allows the researcher to create theories or suggestions.^26,27^ The clinic provides sexual and reproductive health services. As part of the standard of practice, clients are advised on HIV prevention measures, post HIV testing, together with an invitation to commence PrEP. HIV counsellors and nurses assisted with identifying those who agreed to commence PrEP. A brief explanation of the study was provided. Those who agreed to participate were invited to answer a self-administered baseline questionnaire, which sought information on demographics, sexual history, PrEP knowledge, and reasons for initiating PrEP. Participants who were already using PrEP were excluded from the study.

In the current report, early sexual debut was defined as those who had sex for the first time at 15 years and younger. In an age-disparate relationship, the sexual partner was at least 5 years older than the participants. Transactional sex was defined as an exchange of material support for sex. Multiple concurrent partners referred to having two or more sexual partners at the same time.

The questionnaire took approximately 15 min to complete. The researcher or research assistant was present to answer any questions. The questionnaires were available in either English or isiZulu. The baseline questionnaire was completed in a closed clinic room to allow for privacy while completing the questionnaire due to the sensitive nature of some questions. Participants were allowed to withdraw at any point in the study. Verbal cues and body language were taken into consideration, as the study population was predominantly younger; this ensured the participants’ welfare was protected, especially if they did not voice their discomfort. In the current study, none of the participants displayed any signs of discomfort or voiced any questions they may have had. A distress protocol was in place for the relevant referrals for counselling and support groups; however, this was not utilised in the current study.

Prior to the main study, the questionnaires were piloted among PrEP users to ensure reliability and validity. An expert focus group, comprising the researchers, current PrEP users, and nurses (*n* = 6), assessed the validity of the questionnaire, after which minor changes were implemented. Cronbach’s alpha was calculated only for sections comprising grouped items that measured a single construct. These included the abuse sections (type of abuse, α = 0.618; frequency of abuse, α = 0.833), and the PrEP knowledge section (α = 0.612). Other sections of the questionnaire consisted largely of single-item or descriptive measures for which Cronbach’s alpha is not applicable. For further validation, we also computed the Kuder–Richardson 20 coefficient for the dichotomous knowledge items. The Kuder–Richardson 20 value was 0.51, which, while slightly lower than Cronbach’s alpha, is consistent with moderate internal consistency for exploratory research of this nature. A total of 376 questionnaires were completed and included in the analysis.

### Data analysis

All data were analysed using SPSS version 29 (IBM Corp., Armonk, New York, United States). Categorical data were calculated using frequencies, percentages, and standard deviation. Data were correlated using Pearson Chi-Square and Fisher Exact tests to determine bivariate associations. Missing data were excluded from the analyses. Participants with incomplete responses were not excluded from the dataset unless all responses for a construct were missing. In line with SPSS defaults, pairwise deletion was applied for descriptive and reliability analyses, while listwise deletion was used for regression models to ensure only participants with complete data on included predictors were analysed. A *P*-value of < 0.05 was deemed statistically significant.

### Ethical considerations

Ethics approval was obtained from the Durban University of Technology Institutional Ethics Review Committee (reference number: IREC 148/20). Permission from the research clinic site, clinic manager, and the Department of Health, South Africa, was obtained prior to commencing the research. All participation was voluntary, and written informed consent was obtained. The questionnaire was answered in the privacy of a clinic room. Verbal cues and body language were taken into consideration, as the study population was predominantly younger, ensuring the participants’ welfare was protected, especially if they did not voice their discomfort. In the current study, none of the participants displayed any signs of discomfort or voiced any questions they might have.

## Results

The demographic profile and sexual behavioural characteristics of the participants is reported in Table 1. Most participants were Black African (*n* = 376; 100%), single (*n* = 343; 91.5%), and heterosexual (*n* = 368; 98.1%). The mean age of the participants was 30.2 ± 6.99 years. Sexual debut occurred at 18.4 ± 2.58 years. Only 41.7% disclosed that they were in a serodiscordant relationship (*n* = 88). A small proportion (*n* = 76; 20.2%) was previously diagnosed with a sexually transmitted infection. Thirty-one participants (8.3%) reported having been subjected to some form of abuse, with some experiencing more than one type of abuse. Of those, physical abuse (*n* = 5; 16.1%), emotional abuse (*n* = 31; 8.2%), mental abuse (*n* = 1; 3.2%), and sexual abuse (*n* = 1; 3.2%) were identified among the participants. Those who were single and male reported having five or more lifetime partners. Concurrent sexual partners were more commonly reported among male participants than their female counterparts. Age-disparate relationships and transactional sex were reported by women.

**TABLE 1:** Participant characteristics at the point of pre-exposure prophylaxis initiation.

Characteristic	Gender						Age (years)												Relationship status							
	Male		Female		Total		18–24		25–29		30–34		35–39		≥ 40		Total		Single		Divorced		Married		Total	
	*n*	%	*n*	%	*N*	%	*n*	%	*n*	%	*n*	%	*n*	%	*n*	%	*N*	%	*n*	%	*n*	%	*n*	%	*N*	%
**Number of lifetime partners**																										
1 partner	0	-	38	11.6	38	10.2	13	15.1	10	9.8	8	9.3	2	3.3	5	13.2	38	10.2	29	8.6	1	33.3	8	27.6	38	10.2
2 partners	2	4.5	38	11.6	40	10.8	15	17.4	9	8.8	7	8.1	7	11.9	2	5.2	40	10.8	34	10.0	1	33.3	5	17.2	40	10.8
3 partners	2	4.5	48	14.7	50	13.5	13	15.1	14	13.7	11	12.8	8	13.6	4	10.5	50	13.5	47	13.9	0	0.0	3	10.4	50	13.5
4 partners	2	4.5	45	13.8	47	12.7	11	12.8	14	13.7	9	10.5	8	13.6	5	13.2	47	12.7	41	12.1	0	0.0	6	20.7	47	12.7
5 partners and/or more	38	87.0	158	48.3	196	52.8	34	39.6	55	54.0	51	59.3	34	57.6	22	57.9	196	52.8	188	55.4	1	33.4	7	24.1	196	52.8

**Total**	**44**	**100.0**	**327**	**100.0**	**371**	**100.0**	**86**	**100.0**	**102**	**100.0**	**86**	**100.0**	**59**	**100.0**	**38**	**100.0**	**371**	**100.0**	**339**	**100.0**	**3**	**100.0**	**29**	**100.0**	**371**	**100.0**

**Age of current sexual partner (years)**																										
18–24	2	5.0	23	7.5	25	7.2	22	30.6	32	44.4	14	19.4	4	5.6	0	-	72	100.0	24	7.6	1	33.3	0	0.0	25	7.2
25–29	12	30.0	50	16.2	62	17.8	2	2.0	27	27.3	46	46.5	20	20.2	4	4.0	99	100.0	62	19.7	0	0.0	0	0.0	62	17.8
30–34	9	22.5	77	25.1	86	24.8	1	1.2	3	3.7	18	22.2	46	56.8	13	16.1	81	100.0	83	26.3	0	0.0	3	10.4	86	24.8
35–39	10	25.0	85	27.7	95	27.4	0	0.0	0	0.0	8	13.8	22	37.9	28	48.3	58	100.0	90	28.6	0	0.0	5	17.2	95	27.4
40 and older	7	17.5	72	23.5	79	22.8	0	0.0	0	0.0	0	0.0	3	8.1	34	91.9	37	100.0	56	17.8	2	66.7	21	72.4	79	22.8

**Total**	**40**	**100.0**	**307**	**100.0**	**347**	**100.0**	**25**	**7.2**	**62**	**17.9**	**86**	**24.8**	**95**	**27.4**	**79**	**22.7**	**347**	**100.0**	**315**	**100.0**	**3**	**100.0**	**29**	**100.0**	**347**	**100.0**

**Multiple concurrent partners**																										
Yes	24	54.5	38	12.3	62	17.5	9	12.3	22	21.6	14	16.7	10	17.2	7	18.9	62	17.5	61	18.9	0	0.0	1	3.4	62	17.5
No	20	45.5	272	87.7	292	82.5	64	87.7	80	78.4	70	83.3	48	82.8	30	81.1	292	82.5	261	81.1	3	100.0	28	96.6	292	82.5

**Total**	**44**	**100.0**	**310**	**100.0**	**354**	**100.0**	**73**	**100.0**	**102**	**100.0**	**84**	**100.0**	**58**	**100.0**	**37**	**100.0**	**354**	**100.0**	**322**	**100.0**	**3**	**100.0**	**29**	**100.0**	**354**	**100.0**

**Age-disparate relationship**																										
Yes	3	7.5	118	38.4	121	34.9	40	55.6	38	38.4	41	50.6	2	3.4	0	-	121	34.9	116	36.8	0	0.0	5	17.2	121	34.9
No	37	92.5	189	61.6	226	65.1	32	44.4	61	61.6	40	49.4	56	96.6	37	100.0	226	65.1	199	63.2	3	100.0	24	82.8	226	65.1

**Total**	**40**	**100.0**	**307**	**100.0**	**347**	**100.0**	**72**	**100.0**	**99**	**100.0**	**81**	**100.0**	**58**	**100.0**	**37**	**100.0**	**347**	**100.0**	**315**	**100.0**	**3**	**100.0**	**29**	**100.0**	**347**	**100.0**

**Transactional sex**																										
Yes	1	2.2	23	7.0	24	6.5	10	12.2	9	8.6	4	4.5	1	1.7	0	-	24	6.5	23	6.8	0	-	1	3.4	24	6.5
No	44	97.8	304	93.0	348	93.5	72	87.8	96	91.4	84	95.5	58	98.3	38	100.0	348	93.5	317	93.2	3	100.0	28	96.6	348	93.5

**Total**	**45**	**100.0**	**327**	**100.0**	**372**	**100.0**	**82**	**100.0**	**105**	**100.0**	**88**	**100.0**	**59**	**100.0**	**38**	**100.0**	**372**	**100.0**	**340**	**100.0**	**3**	**100.0**	**29**	**100.0**	**372**	**100.0**

Note: Not all percentages add up to 100 because of multiple responses or missing data.

Factors associated with sexual behaviour trends are shown in Table 2. Bivariate analysis indicated that younger participants were more likely to have a sexual partner aged 5 years or older than themselves. Specifically, 55.6% (*n* = 40) of participants aged 18 years to 24 years reported having partners five or more years older. Notably, only 3.4% of participants aged 35 years to 39 years (*n* = 2), and none of those aged 40 years and older, reported having partners at least 5 years or older (Fisher’s Exact test *P* < 0.001; Table 2). More participants aged 18 years to 24 years (*n* = 10; 12.2%) reported engaging in transactional sex compared to those aged 35 years to 39 years (*n* = 1; 1.7%; Fisher’s Exact test *P* < 0.034). None of the participants aged 40 years and older reported transactional sex. Participants who reported sexual debut at the age of 19 years or younger were more likely to have five or more lifetime partners (Fisher’s Exact *P* < 0.001). A linear trend was observed between age of sexual debut and number of lifetime sexual partners (Pearson Chi-Square test *P* < 0.001). Having five or more lifetime partners was reported for those who reported sexual debut at an age younger than 15 years (*n* = 18; 9.3%), or those between the ages of 15 years and 19 years (*n* = 144; 74.2%). However, when sexual debut occurred between the ages of 20 years and 24 years, 42.5% (*n* = 17) reported having only two lifetime partners. Early sexual debut was also positively correlated with age-disparate relationships (Fisher’s Exact test *P* < 0.033).

**TABLE 2:** Factors influencing behavioural trends among the cohort initiating pre-exposure prophylaxis.

Characteristic	Age-disparate relationship					Transactional sex					Sexual debut (years)								
	Yes		No		Total	Yes		No		Total	<15		15–19		20–24		25–30		Total
	*n*	%	*n*	%		*n*	%	*n*	%		*n*	%	*n*	%	*n*	%	*n*	%	
**Participants age (years)**																			
18–24	40	55.6	32	44.4	**72**	10	12.2	72	87.8	**82**	3	16.7	66	26.5	17	18.1	0	0.0	**86**
25–29	38	38.4	61	61.6	**99**	9	8.6	96	91.4	**105**	4	22.2	72	28.9	27	28.7	1	14.3	**104**
30–34	41	50.6	40	49.4	**81**	4	4.5	84	95.5	**88**	7	38.9	54	21.7	21	22.3	3	42.9	**85**
35–39	2	3.4	56	96.6	**58**	1	1.7	58	98.3	**59**	0	-	41	16.5	15	16.0	2	28.5	**58**
40 and older	0	0.0	37	100.0	**37**	0	0.0	38	100.0	**38**	4	22.2	16	6.4	14	14.9	1	14.3	**35**

**Total**	**121**	**34.9**	**226**	**65.1**	**347**	**24**	**6.5**	**348**	**93.5**	**372**	**18**	**100.0**	**249**	**100.0**	**94**	**100.0**	**7**	**100.0**	**368**

	*P*-value < 0.001*					*P*-value < 0.034*					*P*-value < 0.029*								
**Sexual partners age (years)**																			
18–24	0	-	25	100.0	**25**	0	0	25	100	**25**	2	8.0	21	84.0	2	8.0	0	0.0	**25**
25–29	22	35.5	40	64.5	**62**	8	13.6	51	86.4	**59**	3	4.8	43	69.4	16	25.8	0	0.0	**62**
30–34	28	32.6	58	67.4	**86**	6	7.1	79	92.9	**85**	3	3.6	56	66.7	24	28.6	1	1.2	**84**
35–39	52	54.7	43	45.3	**95**	3	3.2	92	96.8	**95**	7	7.5	68	73.1	17	18.3	1	1.1	**93**
40 and older	19	24.1	60	75.9	**79**	5	6.3	74	93.7	**79**	2	2.7	45	60.0	26	34.6	2	2.7	**75**

**Total**	**121**	**34.9**	**226**	**65.1**	**347**	**22**	**6.4**	**321**	**93.6**	**343**	**17**	**5.0**	**233**	**68.7**	**85**	**25.1**	**4**	**1.2**	**339**

	*P*-value < 0.001*					*P*-value = 0.076					*P*-value = 0.240								
**Number of lifetime partners**																			
1 partner	8	21.6	29	78.4	**37**	2	5.6	34	94.4	**36**	0	0.0	23	63.9	12	33.3	1	2.8	**36**
2 partners	9	26.5	25	73.5	**34**	2	5	38	95	**40**	0	0.0	20	50.0	17	42.5	3	7.5	**40**
3 partners	11	25.0	33	75.0	**44**	1	2	49	98	**50**	0	0.0	28	58.3	18	37.5	2	4.2	**48**
4 partners	18	40.0	27	60.0	**45**	2	4.3	45	95.7	**47**	0	0.0	32	68.1	15	31.9	0	0.0	**47**
5 partners or more	72	39.3	111	60.7	**183**	16	8.2	178	91.8	**194**	18	9.3	144	74.2	31	16.0	1	0.5	**194**

**Total**	**225**	**65.6**	**118**	**100.0**	**343**	**23**	**6.3**	**344**	**93.7**	**367**	**18**	**4.9**	**247**	**67.7**	**93**	**25.5**	**7**	**1.9**	**365**

	*P*-value = 0.093					*P*-value = 0.507					*P*-value < 0.001*								

Note: Bivariate data analysis used Pearson’s Chi-Square and Fisher’s Exact tests. Not all percentages add up to 100 because of multiple responses or missing data.

* , a *p*-value of < 0.05 was deemed significant.

Although the relationship between the number of lifetime partners and the likelihood of having a partner aged 5 years or older was not statistically significant (Pearson Chi-Square test *P* = 0.093), a linear-by-linear association indicated a trend between age disparity and number of lifetime partners (*P* = 0.009). Of those who had only one lifetime partner,few (*n* = 8; 21.6%) reported age-disparate relationships, in contrast to 39.3% (*n* = 72) participants with five or more lifetime partners being in age-disparate relationships (Table 2).

## Discussion

The current study explored sexual practices that increased HIV vulnerability amongst primary healthcare clinic clients at the point of PrEP initiation. Early sexual debut, serodiscordant relationships, multiple concurrent sexual partnerships, age-disparate relationships, and transactional sex were identified, particularly amongst younger participants aged 18 years to 24 years. Transactional sex and age-disparate relationships were more common among women and among younger participants. Young people, aged approximately 18 years, are likely to have completed secondary education and are entering a tertiary institution, which may require a move away from home to a new city.^28^ This is often accompanied by stresses of living expenses or concern about their family households’ expenses, leading to relationships with older partners who are financially secure.^29,30^ Such transactional relationships not only pay for the basic needs of these young students but also provide funds for luxuries such as hair and nail salon visits and other extravagant items.^31,32^

Women in transactional relationships are unable to negotiate safe sex, including the use of condoms,^33,34^ thus increasing their vulnerability to HIV infection. When men are perceived as benefactors of resources, it gives them control over sexual relationships.^31^ Furthermore, when a man pays for a restaurant bill, which often includes alcohol, women reciprocate by exchanging sexual favours.^35^ When evaluating immediate benefits, the concept of seroconverting held little significance, as economic gain was prioritised above health considerations.^31^ In addition, peer pressure and social media influence are also drivers for transactional sex among adolescents, as it improves their social status.^10,29,30,36^ This is because of the pressure of social trends to access the current fashionable accessories or other material goods, emphasising the importance of material ownership. Additionally, friends were envious of their lifestyles and felt pressured to maintain their social appearances, which resulted in transactional sex where their male partners were able to finance their desired lifestyle.

A Nigerian study^30^ reported that family pressure for financial support contributed towards engaging in transactional sex. This was supported by a study conducted in Kenya, which reported that these requests for familial support were because of unemployment, with mothers in particular applauding their daughters when they sent money and other items home. As the men were aware of this parental influence, it increased the power imbalance in the relationship.^37^ Financial constraints are contributing factors towards transactional relationships where sexual favours are exchanged for material support. This highlights the cultural norms within relationships, where men make decisions about sexual health, and women are financially reliant on their partners. However, this sexual exchange is confused with affection, further highlighting male dominance and unequal gender dynamics.

The current study population accessed public healthcare facilities, since private healthcare is expensive in South Africa. Only 16.2% of South Africans access private healthcare through private medical schemes.^38^ Our participants were thus from low socio-economic communities, with financial constraints and financial demands by family possibly leading to early sexual debut, particularly when adolescents come from single-parent homes.^39,40^ In this study, sexual debut occurred at a mean age of 18.4 years, with some reporting sexual debut before the age of 15 years. McClinton Appollis et al.^41^ found that 8.9% of adolescent girls and young women aged 15 years to 24 years in six districts in South Africa had sex before the age of 15 years. This is also common in other countries in Africa.^42,43^ Early sexual initiation was disproportionately concentrated among the poor in sub-Saharan Africa.^44^ Surprisingly, being without access to the internet was significantly correlated with early sexual debut in Zambia,^43^ as well as having the ideology that sex was okay even before 18 years.^45^ Peer pressure, societal norms, and substance use also play a role in early sexual debut.^35,46,47^ In contrast, when the family restricts dating, early sexual debut is less likely to occur.^40^ Early sexual debut not only increases the risk of HIV infection and other sexually transmitted infections, but also of unplanned pregnancy and unsafe abortion.^44^

Early sexual debut has further consequences. We report a positive correlation between early sexual debut and age-disparate relationships, as well as a higher number of lifetime sexual partners. Young women with older partners are at a higher risk of engaging in unsafe sexual behaviours, such as unprotected sex and being in relationships with partners living with HIV.^48^ Furthermore, a higher number of sexual partners is another risk factor for HIV acquisition, as it creates many interwoven sexual networks.^49^ Our findings are supported by McClinton Appollis et al.^41^ and Kyei-Arthur, Agyekum and Kyei-Gyamfi,^50^ who also reported the association between early sexual debut and age-disparate relationships. Furthermore, these studies also reported that early sexual debut was associated with a higher number of lifetime partners. Thus, this cycle of transactional sex, age-disparate relationships, and early sexual debut was also identified in the current study as being associated with vulnerability, leading to HIV infection. We recommend that interventions to tackle age-disparate relationships, especially among young females aged 15 years to 24 years, should be tailored towards increasing education about sexual risk behaviours and economic independence.

### Recommendations

Early sexual health education should be implemented within schools and communities, in an attempt to delay sexual debut. Parents, guardians, and caregivers should be included in these programmes to facilitate open and honest conversations about sexual health. Mainstream and social media can portray accurate decisions in sexual relationships and their consequences. Economic support, such as scholarships or stipends, would be vital to young girls and women to mitigate early sexual debut and transactional sex.

### Limitations

Our sample comprised those enrolling for PrEP, thus the reported sexual behaviours may be biased and not representative of the entire population. Furthermore, the responses were self-reported, which could have the potential of under-reporting certain behaviours, such as transactional sex or abuse, for social desirability. Similarly, the missing data could be a result of social desirability bias. The cross-sectional design limits causal inference. Multivariate analysis was not conducted due to missing data and purposive sampling, which would not be beneficial for regression models. Data on sensitive topics such as sexual behaviours can be underreported and affect the validity of multivariate models. Convenience sampling can cause potential bias.

## Conclusion

Sexual practices that increase HIV vulnerability identified in this study allude to participants’ self-awareness of their perceived risk for HIV acquisition. Hence, their exploration of PrEP as an HIV prevention strategy. The study alludes to economic constraints leading to sexual behaviours that may increase vulnerability to HIV. Thus, initiatives that promote financial independence and life skills among adolescent girls and young women can reduce economic dependence on men, decreasing these behaviours that lead to HIV vulnerability. Gender-transformative approaches would be vital in breaking the power disproportionateness, fostering healthier relationship dynamics, and contributing to long-term reductions in HIV incidence through enhanced agency and structural resilience.
